# Retraction: Curcumin improves palmitate-induced insulin resistance in human umbilical vein endothelial cells by maintaining proteostasis in endoplasmic reticulum

**DOI:** 10.3389/fphar.2023.1247749

**Published:** 2023-07-06

**Authors:** Frontiers Editorial Office

Following publication, concerns were raised regarding the integrity of the images in the published figures. The authors failed to provide the raw data or a satisfactory explanation during the investigation, which was conducted in accordance with Frontiers’ policies. Given the concerns about the validity of the data, and the lack of raw data, the editors no longer have confidence in the findings presented in the article.

The authors agree to this retraction.

This retraction was approved by the Chief Editors of Frontiers in Pharmacology and the Chief Executive Editor of Frontiers.

